# Self-Care Behaviors, Health Indicators, and Quality of Life: A Comprehensive Study in Newly Diagnosed Type 2 Diabetes Patients

**DOI:** 10.3390/nursrep15060201

**Published:** 2025-06-04

**Authors:** Emirjona Kiçaj, Aurela Saliaj, Rudina Çerçizaj, Vasilika Prifti, Sonila Qirko, Liliana Rogozea

**Affiliations:** 1 Faculty of Medicine, Transylvania University, 500036 Brasov, Romania; rudina.cercizaj@unitbv.ro (R.Ç.); vasilika.prifti@unitbv.ro (V.P.); sonila.qirko@unitbv.ro (S.Q.); r_liliana@unitbv.ro (L.R.); 2 Faculty of Health, University “Ismail Qemali” Vlore, 9401 Vlore, Albania; aurela.dai@univlora.edu.al

**Keywords:** newly diagnosed diabetic individuals, nursing, quality of life, self-care

## Abstract

**Background:** Type 2 diabetes (T2D) is a chronic disease that significantly impacts individuals’ quality of life, affecting their physical, psychological, social, and environmental well-being. **Objectives**: This study investigates how self-care habits influence quality of life and key health indicators, such as glycated hemoglobin (HbA1c), blood sugar levels, and body mass index (BMI), among newly diagnosed diabetic individuals in Vlore, Albania. **Methods**: In this cross-sectional study, 332 individuals recently diagnosed with diabetes were surveyed between April and July 2024. Data were collected using the World Health Organization Quality of Life Assessment (WHOQOL-BREF) and the Summary Diabetes Self-Care Activity (SDSCA) surveys. Sociodemographic and clinical information, including age, education, occupation, duration of diabetes, HbA1c, and BMI, were collected through structured interviews and medical records. Descriptive and multivariate analyses were conducted to examine the relationships between self-care behaviors, sociodemographic factors, and quality of life. **Results**: The findings reveal a low quality of life, with a mean quality of life (QoL) score of 35.33 ± 8.25. Environmental domains were most affected, registering a low QoL score of 30.93 ± 9.04. Significant relationships between QoL, self-care practices, and sociodemographic factors and pathologic factors were found. The analysis indicated that distinct factors influenced various domains of quality of life. Physical health was associated with residence, comorbidities, BMI, and HbA1c, follow-up visits, dietary self-care and physical activity self-care. Psychological health correlated with residence, educational level, BMI, and HbA1c, follow-up visits, dietary, physical activity and foot self-care. Age, occupation, BMI, and physical activity self-care were linked to social relationships. Finally, environmental well-being was influenced by gender, residence, BMI, HbA1c, follow-up visits, and dietary and physical activity self-care. **Conclusions**: This study emphasizes the impact of sociodemographic and clinical factors on the quality of life of patients with T2D. Older age, lower education levels, comorbidities, increase in BMI and HbA1c levels, and inadequate self-care were associated with reduced quality of life. These findings highlight the need for targeted interventions and policies that promote self-care and support for at-risk groups.

## 1. Introduction

The incidence of type 2 diabetes (T2D) is a global health threat worldwide and one which is growing at an alarming pace, contributing to the economy and society [1,2]. Disease management is key to preventing complications, and it almost invariably requires a mix of medications and lifestyle changes [3]. Well-organized diabetes care is essential, especially in the initial stage [4,5]. Studies have shown that initiating preventive action early on can be pivotal in people’s ability to manage blood sugar levels, affecting their long-term health outcomes [6,7]. Diabetes significantly reduces the quality of life, affecting physical, mental, and social well-being [8,9]. It impairs a person’s daily functioning and overall well-being. Quality of life refers to how individuals feel physically and mentally and function in their social and personal lives. Factors, like age, emotional health, and the support of family and friends can all influence quality of life [10]. Managing diabetes effectively—through a balanced diet, regular exercise, and proper medication—can help individuals feel better and maintain their health over time [11]. By effectively taking control of their condition, patients improve their physical health and enhance their overall enjoyment of life [12]. Little is known about self-management practices and quality of life among individuals newly diagnosed with T2D in Albania. Although prior studies have examined self-care behaviors and quality of life independently, few have explored how these factors interact with clinical outcomes, particularly in early-stage T2D patients. This study addresses this gap by focusing on individuals newly diagnosed with T2D who received care at healthcare centers in Vlore, Albania. We aim to assess the relationships between self-care activities, QoL, and key clinical indicators, such as HbA1c, blood glucose levels, and BMI. Understanding self-care behaviors, quality of life, and clinical factors is essential for managing diabetes [13]; by mapping these relationships, our findings could offer new insights to enhance the early management of diabetes in Albania.

Self-care, including healthy eating, physical activity, medication adherence, and blood glucose monitoring, is a cornerstone of diabetes management, yet its specific links to clinical and psychosocial outcomes remain understudied in newly diagnosed populations. Eating a healthy diet, engaging in regular physical activity, and monitoring blood sugar level are examples of self-care behaviors with evidence-based associations with improved health outcomes [14]. By mapping these connections, our study seeks to provide evidence that can inform tailored strategies to improve patient outcomes during the critical early phase of disease management. Therefore, this study aims to evaluate the impact of self-care practices on clinical outcomes and quality of life to inform the development of diabetes care and education strategies, particularly during the early stages of the disease in Albania.

The research questions guiding this study are as follows:

- What are the self-care practices and quality of life levels among patients newly diagnosed with T2D in Vlore, Albania?
- What is the relationship between clinical and sociodemographic factors and quality of life among newly diagnosed T2D patients?
- What is the relationship between self-care practices and quality of life among newly diagnosed T2D patients?

## 2. Materials and Methods

### 2.1. Study Design

A cross-sectional study was conducted using the WHOQOL-BREF and the SDSCA questionnaires to assess the quality of life and self-care behaviors of newly diagnosed diabetic patients.

### 2.2. Study Setting

Data for this study were collected from patients attending primary healthcare centers in Vlora from April to July 2024. The selection of these centers is appropriate, as they represent the main point of contact for patients with type 2 diabetes, enabling data collection from a representative sample. Furthermore, these centers play a key role in patient monitoring and health education, providing a suitable context for assessing self-care and quality of life.

### 2.3. Participants

This study used the following cross-sectional descriptive formula to determine the sample size:

$$N=\frac{Z^{2}\cdot p\cdot (1-p)}{d^{2}}$$

where

- *N*—minimum sample size.
- *Z*—standard deviation of 1.96 for a 95% confidence interval.
- *p*—incidence of diabetes in Albania (10% or 0.10).
- *d*—precision or acceptable error, set at 0.05.

The calculation resulted in a sample size of 138 individuals for this study. However, 332 patients with T2D, diagnosed for less than a year and receiving healthcare in primary care centers in Vlora, were included. Selecting a larger sample allowed for the inclusion of a more diverse and representative group, enhancing the accuracy and reliability of the results. This adjustment was made to account for potential non-responses and dropouts, as well as to enhance the robustness of the findings. The additional participants were invited beyond the original estimate to ensure a more representative and diverse sample. Invitations were extended through health centers in Vlore, where participants were selected based on inclusion criteria related to newly diagnosed T2D patients. The sampling method was purposive, focusing on individuals who met specific criteria, such as being newly diagnosed and receiving care at these health centers.

Specific inclusion and exclusion criteria were applied to ensure that the study focused on a clinically relevant and manageable population. The age range of 18–75 years was chosen to reflect the adult population most commonly diagnosed with T2D. Individuals under 18 were excluded due to the different characteristics and treatment approaches of T2D in younger patients. Similarly, those over 75 were excluded to minimize the influence of comorbidities, cognitive decline, or functional limitations, which could affect self-care practices and complicate the interpretation of results.

Only patients diagnosed with T2D within the past 12 months (i.e., 0–12 months prior to enrollment) were included, to ensure the study focused on individuals in the early stages of the disease. The study was limited to those receiving care at primary healthcare centers in Vlora, as this setting was central to the research scope. Participants were required to have sufficient physical and mental capacity to engage meaningfully with the study procedures, even if they had other health conditions. Furthermore, it was necessary that participants spoke the language of the study and could understand the study materials, to ensure reliable data collection. These criteria were designed to promote a homogeneous sample while reflecting real-world clinical diversity. The complete list of inclusion and exclusion criteria is presented in Table 1.

### 2.4. Data Sources/Measurement: Several Instruments Were Used for This Study

Part One: Sociodemographic Data and Health Status of Diabetic Patients

- Sociodemographic data: age, gender, marital status, religious belief, employment status, level of education, and place of residence (urban/rural).
- Health characteristics: duration of diabetes; BMI (at the time of diagnosis and at the time of interview); weight (at the time of diagnosis and at the time of interview); family history of diabetes (yes/no) and degree of closeness; blood pressure; blood glucose level (at the time of diagnosis and at the time of interview); HbA1c level (at the time of diagnosis and at the time of interview); lipid profile; presence of comorbidities; and alcohol and tobacco use.

Part Two: Quality of Life Measurement

The standardized version of WHOQOL-BREF, developed and distributed by the World Health Organization, headquartered in Geneva, Switzerland (Avenue Appia 20, 1211 Geneva, Switzerland), was utilized to assess the quality of life of diabetic patients [15]. Among the twenty-six questions in the survey, the first two pertain to the overall quality of life, seven questions address the physical domain, six relate to the psychological domain, three emphasize social support, and eight focus on environmental assessment. The questionnaire employs a five-point Likert scale, with inverted responses for questions 3, 4, and 26. The total scores for each domain are converted to a range from 4 to 20 and then adjusted to a scale of 0 to 100.

Part Three: The Summary of Diabetes Self-Care Activities is a validated instrument developed by Dr. Debra J. Toobert and colleagues at the Oregon Research Institute [16], used to assess the frequency of self-care behaviors in patients with diabetes. The questionnaire includes multiple items that evaluate key aspects of diabetes management, including diet, physical activity, glucose monitoring, foot care, and medication adherence. It is designed to capture the extent to which individuals engage in these essential behaviors, which are crucial for managing T2D. The SDSCA has been translated, culturally adapted, and validated in the Albanian language [17], ensuring its applicability in our study population. The instrument includes 11 items, each measuring the frequency of behavior in the past week, with responses on a 0–7 scale, where 0 indicates no activity and 7 indicates daily activity. This allows us to quantify self-care practices and assess their relationship with health outcomes, like HbA1c, blood glucose levels, and BMI.

### 2.5. Variables and Statistical Methods

Two standardized questionnaires collected information on quality of life and self-care practices. Quality of life was assessed using the WHOQOL-BREF questionnaire, based on the standardized 0–100 scoring scale. The average scores are calculated from participants’ responses across each domain of quality of life. The official WHOQOL-BREF user manual and scoring [18], recognized as the international standard for the transformation and interpretation of quality of life scores, were strictly followed in this study.

The independent variables related to quality of life include sociodemographic factors (age, gender, residence, education, employment, marital status, and religious beliefs) and clinical factors (BMI, glycemic levels and changes, presence of comorbidities, smoking and alcohol use, medical check-ups, and patients’ self-care behaviors).

For the statistical analysis, the following aspects were considered:

- Descriptive statistics (mean and standard deviation) were used to describe quantitative variables (e.g., age, BMI, HTN, glycemia, HbA1c, cholesterol, low-density lipoprotein (LDL), high-density lipoprotein (HDL), alcohol/tobacco use, self-care days, and QoL scores
- Frequency (percent) was utilized for qualitative variables.
- The interpretation of variables also included calculating changes in HbA1c levels and BMI from the time of diagnosis to the time of study inclusion.
- As the data did not follow a normal distribution (as verified by normality test), non-parametric tests were applied.

Statistical methods were stratified by research questions, as follows.

Section 3.2 and Section 3.3 of the results address research question 1, i.e., “What are the self-care practices and quality of life levels among patients newly diagnosed with T2D in Vlore, Albania?”. Descriptive statistics (means, SDs, and frequencies) were used to report self-care activity levels across domains (diet, physical activity, blood glucose monitoring, foot care, and medication adherence).

Section 3.1 and Section 3.4 of the results address research question 2, i.e., “What is the relationship between clinical and sociodemographic factors and quality of life among newly diagnosed T2D patients?”. For this research question, the following methods were used:

- Mann–Whitney U test for variables with two categories.
- Kruskal–Wallis test for variables with more categories, followed by Dunn’s post hoc test with Bonferroni correction when significant differences were found.

Section 3.5 of the results addresses research question 3, i.e., “What is the relationship between self-care practices and quality of life among newly diagnosed T2D patients?”. The following methods were used:

- Simple linear regression assessed the relationship between overall self-care and each QoL domain.
- Multiple linear regression was performed to examine the influence of factors on overall QoL.
- The significance level was set at *p* ≤ 0.05, and multicollinearity was checked using VIF, which was acceptable (VIF ≤ 1.7).

Additionally, variables with more than two categories were coded into the following binary variables:

- Employment (employed/unemployed, which includes unemployed and retired).
- Education (≤higher, incomplete primary, primary, high school, tertiary education).
- Civil status (Married/unmarried, which includes single, divorced, and widowed).
- Duration of illness (<6 months: <3 months, 3–6 months/≥6 months: 6–9 months, 9–12 months).
- Visits to a specialist doctor (Never or once per year/more often: two or more times per year).

Self-care was categorized as “Active” (average days of self-care > 3 days per week) or “Inactive” (average days of self-care ≤ 3 days per week). The Cronbach’s alpha value of 0.77 indicates acceptable internal consistency (reliability) for the WHOQOL-BREF and SDSCA scales.

The data were analyzed using IBM SPSS Statistics for Windows, Version 23.0 (Armonk, NY, USA: IBM Corp.). More information about the software can be found at https://www.ibm.com/products/spss-statistics (accessed on 10 January 2024). 

### 2.6. Ethical Considerations

This study was conducted following the ethical principles of scientific research, adhering to the Helsinki Code to safeguard the rights of individuals participating in research. The study implementation received approval from the Scientific Ethics Committee at the Faculty of Health, “Ismail Qemali” University, under approval number 80/4. Necessary permissions were obtained for the use of the study instruments, and written authorization for conducting the study was secured from the local health authorities of the Vlora municipality.

The study participants were fully informed, and participation was voluntary. All participating patients provided written informed consent and could withdraw from the study at any time without any impact on their future treatment.

## 3. Results

A total of 341 questionnaires were distributed as part of the study. Of these, 332 were fully completed and met the inclusion criteria, providing complete and accurate data for analysis.

### 3.1. Sociodemographic Data and Pathological Profile of Study Participants

Table 2 presents the sociodemographic characteristics and pathological profiles of the participants. The mean age of the patients was 66.7 ± 9.1 years, with an age range of 41 to 75 years. Among the participants, 52.7% were female, 88% were married, 53% had a secondary education level, 82.8% lived in an urban area, and 65% were retired.

The largest group of participants (34.9%) had a disease duration of 6 to 9 months. Only 18.7% reported a family history of diabetes, with 16.6% having a first-degree relative with diabetes.

When comparing their status at diagnosis to the time of the study, a slight decrease in the number of patients classified as overweight or obese was observed. Additionally, a reduction in average glycemia levels was noted from the time of diagnosis to the moment of the interview (baseline glucose: 237.7 ± 71.5 mg/dL vs. current glucose: 184.4 ± 62.7 mg/dL). A similar trend was observed in HbA1c levels, decreasing from 8.6 ± 1.6% at baseline to 8.0 ± 1.4% at the interview.

Furthermore, 48.2% of participants had at least one comorbid condition, with HTN present in approximately 43.7% of cases. Alcohol consumption was reported by 4.2% of participants, with an average duration of 12 ± 9.3 years and an average daily intake of 29.3 ± 24.9 mL. Smoking was reported by 5.1% of patients, with an average smoking duration of 12.6 ± 7.8 years and an average daily consumption of 6.2 ± 5.3 cigarettes.

**Figure 1:**
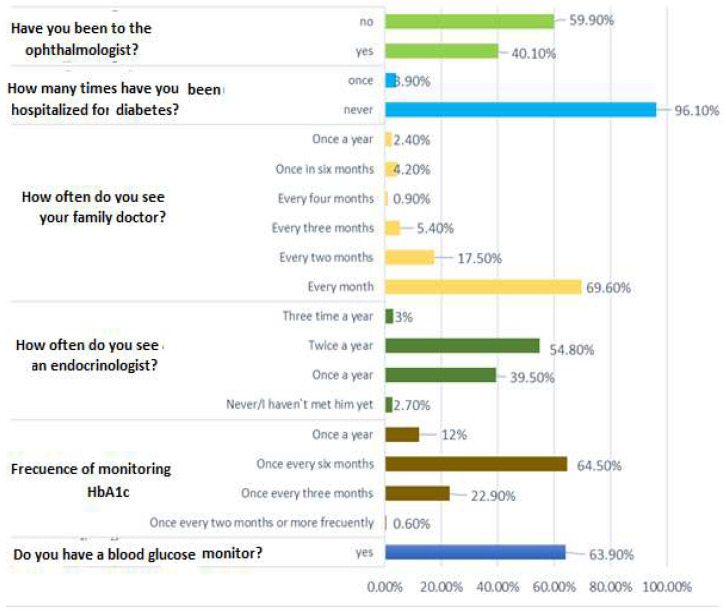
Use of health equipment and services by participants.

Figure 1 illustrates the use of health equipment and services among diabetic patients included in the study. Here, 63.9% of patients reported owning a personal glucometer. Regarding HbA1c monitoring, 64.5% of patients indicated measuring it every six months, 12% once a year, and 22.9% every three months. Regarding specialist visits, around 39.5% reported seeing an endocrinologist once a year, while 2.7% had never consulted one. Additionally, 69.6% of patients noted visiting their family doctor monthly. However, only 40.1% of patients had seen an ophthalmologist.

### 3.2. Self-Care Practices of Newly Diagnosed Diabetic Patients

Self-care practices among newly diagnosed diabetic patients were evaluated using the SDSCA questionnaire, as detailed in Table 3. A noticeable trend is that patients showed the highest adherence to medication use, averaging 5.5 ± 0.75 days per week. In contrast, dietary care was practiced for an average of 3.4 ± 0.79 days per week. Physical activity was reported for an average of 2.5 ± 1.25 days per week, while glycemia measurement and foot care were less frequent, with averages of 1.5 ± 0.90 and 1.4 ± 0.60 days per week, respectively. Among patients who smoked, the average number of cigarettes smoked per day decreased from 15.3 ± 8.1 at the time of diagnosis to 6.2 ± 5.3 after the initiation of treatment.

### 3.3. Using WHOQOL-BREF to Assess the Quality of Life of Newly Diagnosed Diabetic Patients

According to the WHOQOL questionnaire in Table 4, the quality-of-life assessment results highlight the impact of diabetes diagnosis on patients’ lives. The average scores for the key categories were as follows: physical health (35.36 ± 9.48), psychological health (34.26 ± 9.23), social relationships (40.77 ± 15.93), and environment (30.93 ± 9.04). These findings indicate that diabetes significantly affects all aspects of patients’ lives, with the lowest scores observed in psychological health and environment, reflecting a considerable burden in these areas.

In particular, physical health significantly impacts patients, indicating the substantial difficulties that they experience with their physical abilities, especially in terms of “Work capacity” (21.54 ± 19.99). The psychological health domain’s most noticeable effect was “Thinking, learning, memory, and concentration” (15.36 ± 17.20), suggesting that diabetes has created numerous concerns.

Similarly, in social relationships, patients encountered more significant challenges with “Sexual activity” (18.46 ± 22.49), although it should be noted that most participants in the study were over 60 years old. In the environmental domain, the most significant impact was observed in “Physical freedom and safety” (25.98 ± 6.80), which may reflect the various limitations associated with managing diabetes.

Conversely, some more positive categories exhibited higher scores. Patients gave higher scores in such areas as “Activities of daily living” (43.15 ± 12.75), “Spirituality/Religion/Personal beliefs” (42.84 ± 13.45), “Social support” (49.59 ± 17.20), “Personal relationships” (46.24 ± 18.23), and “Health and healthcare access and quality” (35.39 ± 25.21).

These findings suggest that, while negative impacts are evident in physical and psychological health, certain aspects of social life and support remain relatively strong, indicating that patients can rely on some sources of support and confidence to cope with the disease.

### 3.4. Factors That Influence the Quality of Life of Newly Diagnosed Diabetic Patients

The factors that affect the quality of life for diabetic patients are multifaceted, encompassing various aspects of their lives (Table 5). In the physical health domain, a statistically significant relationship was found with residency (*p* = 0.003). Residents of rural areas reported a lower quality of life concerning physical health.

For the psychological health component, significant associations were identified with education level (*p* = 0.004) and residency (*p* = 0.002), where patients with lower education levels and those living in rural areas experienced a diminished quality of life in this domain. Regarding social support, statistically significant relationships were found with age (*p* < 0.0001) and employment status (*p* = 0.001). Older patients and unemployed individuals reported reduced social support.

In the environmental domain, significant associations were observed with gender (*p* = 0.003) and residency (*p* = 0.001). Women and rural residents reported a lower quality of life in this aspect.

For the overall quality of life (Overall QoL), significant relationships were identified with age (*p* = 0.003). Older individuals exhibited a lower overall quality of life.

Table 6 provides a detailed analysis of how clinical factors and the use of health equipment and services impact patients’ quality of life, revealing crucial associations that could inform improvements in disease management.

The presence of comorbidities negatively influenced the physical (*p* < 0.0001) aspects. Additionally, patients with hypertension (HTN) reported a lower quality of life in the physical (*p* = 0.001) domains.

Notably, changes in BMI levels were associated with a poorer quality of life across all domains (*p* < 0.0001 and *p* = 0.001). Patients showing increased BMI faced more significant challenges. Also, patients with a reduction in HbA1c levels had a better quality of life than those with increased/no change in HbA1c levels in physical, psychological, and environmental domains, and overall (*p* < 0.0001). Moreover, those whose HbA1c reduction reached levels below 7% had a better quality of life than patients with HbA1c levels ≥ 7%, in physical health, psychological health, environmental, and overall domains (*p* < 0.0001).

Visits to specialist doctors affect the quality of life of diabetic patients. Thus, patients who had no visits or fewer visits to the endocrinologist had a lower quality of life in the environmental domain (*p* = 0.003). Those with few or no visits to the ophthalmologist had lower quality of life in the physical (*p* =0.001), psychological (*p* = 0.001), and overall domains (*p* < 0.0001).

### 3.5. Assessment of the Impact of Self-Care on the Quality of Life of Newly Diagnosed Diabetic Patients

To further enhance the analysis, the relationship among different components of quality of life and self-care practices was examined (Table 7).

The results demonstrate a strong statistical association between quality of life and the level of self-care across several crucial aspects of diabetes management. Patients who exhibited lower engagement in overall self-care and physical exercise reported notably lower quality of life across all domains (*p* < 0.0001, *p* = 0.001), suggesting that active participation in these self-care practices is vital for improving overall well-being.

Patients with lower adherence to dietary recommendations also reported significantly lower quality of life in the physical, psychological, environmental, and overall well-being domains (*p* < 0.0001).

Notably, foot self-care had a significant impact on psychological health. Patients who were less active in this area reported lower scores in psychological health (*p* < 0.0001). This suggests that regular foot care may positively influence psychological health.

A multiple linear regression analysis assessed the combined effect of patient characteristics and self-care behaviors on quality of life. The aim was to identify the most significant predictors of changes across its various domains (Table 8).

The analysis revealed that several sociodemographic and health-related characteristics significantly influenced the physical component of quality of life in newly diagnosed patients with T2D. Specifically, comorbidities were associated with poorer physical quality of life by 4.32 points compared to patients without comorbidities (β = −4.32, 95% CI: −6.30 to −2.35; *p* < 0.0001). Similarly, patients who experienced a BMI increase of more than 1% reported poorer physical quality of life by 3.04 points compared to those who had no change or a reduction in BMI (β = −3.04, 95% CI: −4.67 to 1.41; *p* < 0.0001). In addition, an increase in HbA1c was linked to a decrease in physical quality of life by 5.28 points when compared to patients whose HbA1c remained stable or decreased (β = −5.28, 95% CI: −7.48 to −3.09; *p* < 0.0001). Furthermore, patients who had not visited an ophthalmologist reported a poorer physical quality of life by 2.83 points compared to those who had attended at least one visit with this specialist (β = −2.83, 95% CI: −4.82 to −0.84; *p* = 0.005).

In the psychological domain of quality of life, several factors were significantly associated with poor outcomes among newly diagnosed patients with T2D. Patients who experienced an increase in BMI greater than 1% reported a poorer psychological quality of life by 2.28 points compared to those with no change or decrease in BMI (β = −2.28, 95%: −3.90 to −0.66; *p* = 0.004). Similarly, an increase in HbA1c was associated with a 4.59-point decrease in psychological quality of life (β = −4.59, 95% CI: −6.76 to −2.41; *p* < 0.0001). Additionally, those who had not visited an ophthalmologist reported a lower psychological quality of life by 2.59 points compared to those who had attended such visits (β = −2.59, 95% CI: −4.56 to 0.64; *p* = 0.01).

The results in the social relationships domain indicate that various health and self-care factors were linked to a lower quality of life. Patients over 60 reported significantly poorer social quality of life than those under 60, with a difference of 6.61 points (β = −6.61, 95% CI: −11.63 to −1.59; *p* = 0.010). Employment status also played a role; unemployed or retired patients had significantly lower social relationship scores than employed ones, by 6.54 points (β = −6.54, 95% CI: −11.55 to −1.52; *p* = 0.011). Furthermore, an increase in BMI (from the time of diagnosis to the moment of inclusion in the study) greater than 1% was associated with a decrease of 5.12 points in the social domain compared to patients whose BMI remained stable or decreased (β = −5.12, 95% CI: −7.85 to −2.38; *p* < 0.0001).

In the environmental domain, sociodemographic, health, and self-care factors emerged as significant predictors of poorer quality of life. Female patients reported significantly lower environmental quality of life than males, with a difference of 2.49 points (β = −2.49, 95% CI: −4.34 to −0.64; *p* = 0.008). Unemployed individuals reported lower scores than employed individuals by 3.41 points (β = −3.41, 95% CI: −6.02 to −0.80; *p* = 0.011). Similarly, an increase in BMI of more than 1% was linked to a 2.54-point reduction in environmental quality of life (β = −2.54, 95% CI: −4.15 to 0.92; *p* = 0.002).

Increased HbA1c was also associated with a significant decrease of 4.05 points in environmental scores (β = −4.05, 95% CI: −6.24 to −1.85; *p* < 0.0001).

Age, BMI, and HbA1c changes emerged as significant predictors of poorer general quality of life. Patients over 60 had a lower overall quality of life by 4.36 points compared to those under 60 (β = −4.36, 95% CI: −6.29 to 2.42; *p* < 0.0001). Patients with a BMI increase of more than 1% had poorer overall quality of life by 3.33 points compared to those with stable or reduced BMI (β = −3.33, 95% CI: −4.74 to −1.93; *p* < 0.0001). Moreover, an increase in HbA1c was associated with a reduction of 4.75 points in overall quality of life (β = −4.75, 95% CI: −6.67 to −2.84; *p* < 0.0001).

Table 9 presents a multiple linear regression analysis assessing the relationship between various self-care behaviors and different domains of quality of life in newly diagnosed patients with T2D.

The findings demonstrate that overall engagement in self-care activities was significantly associated with better QoL across all domains. Among specific self-care components, dietary management showed a significant positive association with the physical, psychological, environmental, and overall QoL domains, but not with the social relationships domain.

Patients who were not active in dietary self-care reported significantly lower physical QoL scores by 4.74 points (β = −4.74, 95% CI: −6.78 to −2.72; *p* < 0.0001), psychological QoL by 3.63 point (β = −3.63, 95% CI: −5.57 to −1.70; *p* <0.0001), environmental QoL by 3.73 points (β = −3.73, 95% CI: −5.67 to −1.79; *p* < 0.0001), and overall QoL by 3.82 points (β = −3.82, 95% CI: −5.55 to −2.08; *p* <0.0001).

Physical activity was the self-care component with the strongest and most consistent positive association across all domains of QoL. Patients who were not physically active had significantly lower physical QoL scores by 4.32 points (β = −4.32, 95% CI: −6.66 to −1.98; *p* < 0.0001), psychological QoL by 3.35 points (β = −3.35, 95% CI: −5.64 to −1.06; *p* = 0.004), social relationships by 9.27 points (β = −9.27, 95% CI: 13.25 to −5.30; *p* < 0.0001), environmental QoL by 4.80 points (β = −4.80, 95% CI: −7.03 to −2.55; *p* < 0.0001), and overall QoL by 5.45 points (β = −5.45, 95% CI: −7.44 to −3.46; *p* < 0.0001).

Table 10 presents the results of a simple linear regression analysis examining the relationship between total self-care and quality of life domains.

The analysis reveals that total self-care, as a composite measure of all self-care components, has a significant positive impact across all QoL domains. Patients who were not actively engaged in overall self-care reported significantly lower QoL scores than those who were active. Specifically, they scored 7.05 points lower in the physical domain (β = −7.05, 95% CI: −9.51 to −4.58; *p* < 0.0001), 9.25 points lower in the psychological domain (β = −9.25, 95% CI: −11.49 to −6.99; *p* < 0.0001), 7.01 points lower in the relationships domain (β = −7.01, 95% CI: −11.27 to −2.73; *p* = 0.001), 7.15 points lower in the environmental domain (β = −7.15, 7.15, 95% CI: −9.48 to −4.81; *p* <0.0001), and 7.65 points lower in overall quality of life (β = −7.65, 95% CI: −9.74 to −5.55; *p* < 0.0001).

These findings emphasize the critical role of fostering active participation in self-care, particularly regarding diet and physical activity, as essential elements for enhancing the quality of life in individuals newly diagnosed with diabetes.

## 4. Discussion

This study collected data from 332 participants, providing an in-depth look at the health and quality of life of new patients with T2D. The sociodemographic and pathological data analysis highlighted several key factors influencing these patients’ self-care practices and overall quality of life.

The study participants had an average age of 66.7 years, with more than half being women (52.7%). Most participants were married (88%) and had completed secondary education (92%). A significant portion (82.8%) lived in urban areas, and 65% were retired. These findings suggest that individuals with T2D in Albania tend to be elderly, retired, and have an average level of education, resulting in a lifestyle primarily associated with urban environments. These sociodemographic factors play a crucial role in patients’ self-care levels. In a study conducted in Albania, it was found that patients with T2D, particularly the elderly and those with lower levels of education, demonstrated more limited self-care practices, which negatively impacted the overall management of the disease [17]. This aligns with other studies that indicate that type 2 diabetes predominantly affects older adults, particularly in urban areas, where sedentary lifestyles and unhealthy diets may significantly contribute to the condition [19,20]. The high number of retired patients highlights the need for self-care interventions that address age-related challenges, such as physical disabilities and the necessity for social support.

Regarding the pathological history, over half of the patients (48.2%) had a comorbid condition, with hypertension (HTN) being the most common (43.7%). This coexistence of diabetes and other chronic diseases highlights the complexity of managing these patients’ health and underscores the need for an integrated healthcare approach. The observed reduction in glucose and HbA1c levels from diagnosis to the interview (from 237.7 ± 71.5 mg/dL to 184.4 ± 62.7 mg/dL for glucose and from 8.6 ± 1.6% to 8.0 ± 1.4% for HbA1c) indicates a slight improvement in condition management, likely due to treatments and healthcare interventions. This finding aligns with other studies showing that, despite patients’ motivation to enhance self-care, they often encounter challenges in achieving complete glycemic control due to barriers, such as inadequate education and a lack of professional support [19,21].

An interesting finding from the analysis of self-care practices is that patients demonstrated relatively high adherence to medication use, averaging 5.4 days per week.

However, adherence to diet and physical activity was less frequent. Diet was followed on an average of 3.4 days per week, physical activity was performed an average of 2.5 days per week, blood glucose monitoring occurred only 1.5 days per week, and foot care was practiced for 1.4 days per week. This suggests that while patients enhance their diabetes management, certain aspects of self-care, particularly those requiring ongoing commitment, remain underdeveloped. Kamberi et al. found similar trends in Albania, where patients showed varying levels of adherence to self-care practices, underscoring the need for targeted interventions [22]. These findings align with those of Mogre et al., who also observed better adherence to medication than diet and physical activity, highlighting the need for greater support in these areas because of their significant impact on long-term diabetes control [23].

An important finding is the reduction in the number of cigarettes smoked, decreasing from an average of 15.3 to 6.2 cigarettes per day after diagnosis. This encouraging result aligns with studies showing that diabetic patients are highly motivated to quit harmful habits when aware of their negative impact on disease management [24,25]. This behavioral improvement also provides strong evidence of the success of educational interventions and the role of physicians in enhancing patient awareness.

Our study aimed to assess the quality of life (QoL) among newly diagnosed patients with T2D and the factors influencing it. In the studied population, the mean QoL score for patients with diabetes was 35.33 ± 8.25, indicating a low quality of life. This result underscores the significant impact of diabetes on various aspects of patients’ daily lives, including physical, psychological, and environmental domains. The low scores suggest challenges in managing the symptoms and consequences of the disease. These findings highlight the necessity for personalized treatments and ongoing support to enhance quality of life. The variability in results (±8.25) indicates that some patients face more significant challenges, emphasizing the importance of providing individualized support.

The results of the WHOQOL-BREF assessment revealed significant impacts of diabetes on all areas of patients’ lives. Physical abilities were affected, with a low mean QoL score of 21.54 ± 19.99 in “Work capacity”. Additionally, psychological distress was reported as a significant challenge, with a mean score of 15.36 ± 17.20 for “Thinking, learning, memory and concentration” in the psychological domain. Social relationships were affected with a low mean QoL score of 18.46 ± 22.49 in “Sexual activity” and environmental with a low mean QoL score of 25.98 ± 6.80 in “Freedom, physical safety, and security”. However, some areas, such as “Activities of daily living”, “Social support”, and “Personal relationships” showed that patients still had resources to rely on, with higher mean scores in these categories (43.18 ± 12.75, 49.59 ± 17.20, and 46.24 ± 18.23, respectively).

In analyzing factors affecting the physical health component of QoL in patients with T2D, our findings indicate that both sociodemographic and health-related factors significantly influence QoL. Patients living in rural areas are more likely to experience lower physical health scores. Additional factors, such as comorbidities, HTN, increased BMI, elevated HbA1c levels, and the lack of ophthalmologic visits, also negatively affect physical health. These findings are consistent with those of John et al., who reported that BMI is closely linked to QoL, with patients undergoing intensive treatment or facing complications showing lower QoL scores. Similarly, our study emphasizes the negative impact of BMI and comorbidities on physical quality of life [26].

Consistent with the existing literature, our study found that education and residence impact patients’ psychological quality of life, with a low mean QoL score. Patients from rural areas and those with a low level of education are more likely to report a lower psychological quality of life. These findings may be attributed to the pressures associated with a new diagnosis. Additionally, patients with an increase in BMI and HbA1c levels exhibited a lower psychological quality of life, underscoring the importance of disease control in supporting patients’ well-being. Sociodemographic factor, such as age and occupation influence the domain of social relationships. Patients over 60 and those unemployed reported lower quality of life scores in this domain. An increase in BMI was also associated with poorer social relationships.

In the environmental domain, lower QoL scores were observed among female patients, those living in rural areas, individuals with elevated BMI and HbA1c levels, and those who reported infrequent or no visits to an endocrinologist.

Participants who were inactive in self-care activities demonstrated significantly lower quality of life across all domains. In particular, those not engaged in dietary self-care had lower Qol scores in the physical, psychological, and environmental domains.

Furthermore, participants who were inactive in foot care had a lower psychological quality of life.

Several studies suggest that the stress of managing illness and psychological distress can negatively affect patients’ overall experience, including their quality of life, mainly when influenced by such factors as age, health status, and disease treatment. This highlights the need for a more comprehensive understanding of patients’ psychological health [27,28].

Regarding quality of life, the study results show that sociodemographic and self-care factors significantly impact quality of life. Patients over 60 years old and those with lower education levels are more likely to report a lower quality of life. This corresponds with the findings of Wróblewska et al., where age and education level were also linked to a reduced quality of life [29]. These findings are further supported by evidence from Albania, where a study highlighted that collaborative engagement between patients and healthcare professionals notably enhanced self-care behaviors, including tobacco cessation [30]. Furthermore, patients with high BMI and HbA1c levels and those inactive in self-care and physical exercise tend to experience a lower quality of life, highlighting the importance of managing these factors to improve social support and overall quality of life. These results underline the necessity for health interventions that tackle these elements to enhance social support and quality of life in patients with type 2 diabetes.

Consistent with the study by Wróblewska et al., sociodemographic and health factors significantly impact quality of life, particularly in the environmental domain. Our findings indicate that patients over 60, those with hypertension, and those living in rural areas tend to report a lower environmental quality of life. This aligns with Wróblewska et al.’s study, which observed that patients’ location and education level influence their perception of quality of life and the environment [29]. Furthermore, patients who do not participate in self-care activities are more likely to experience lower environmental quality, underscoring the importance of educational interventions and health support to improve living conditions and environmental management. This emphasizes that enhancing quality of life in the environmental domain requires providing patients with opportunities to develop self-care skills and engage in activities that promote a healthier, more supportive environment.

Another important aspect highlighted in this discussion is that interventions aimed at improving education and providing opportunities for social and physical activity can significantly enhance the quality of life for patients with diabetes. Our data indicate that sociodemographic factors, such as age and education level, considerably impact quality of life. This suggests that older patients with lower educational attainment may face more significant challenges when participating in activities that promote their well-being. Furthermore, self-care practices, such as regular physical exercise and a healthy diet, contribute positively to quality of life. Patients not engaging in these activities are likelier to experience a diminished quality of life. This underscores the necessity of promoting health education, supporting patients in making healthy choices, and encouraging participation in activities that can boost their well-being, ultimately leading to an improved quality of life. In a study conducted in Albania, diabetes education during a structured program significantly improved patients’ quality of life and ability to manage the condition. This further supports the critical role of educational interventions [31].

### Strengths and Limitations 

This study offers important insights into the relationship between self-care behaviors and quality of life in individuals newly diagnosed with T2D in Vlore, Albania. One of the strengths of this research is the adequate sample size, which is representative of the newly diagnosed diabetic population in Vlore. We included more patients than the minimum sample size recommended by the formula, ensuring a more reliable and robust data analysis.

Moreover, the study employed a comprehensive self-care questionnaire and reliable statistical methods that identified significant associations between self-care and quality of life, providing valuable insights for healthcare practitioners working with similar populations.

Additionally, the study’s focus on a specific geographic area provides valuable insights into the diabetes management and self-care behaviors specific to Vlore, a region with distinct socioeconomic characteristics. This allows for a deeper understanding of how local factors may influence self-care practices and quality of life outcomes in this population.

While the study’s cross-sectional design provides helpful associations, it does not allow for conclusions about causality. Longitudinal studies would be required to establish causation between self-care behaviors and quality of life. Another limitation is the use of self-reported data, which can be prone to recall bias and social desirability bias, as participants may either overestimate their adherence to self-care behaviors or provide responses that they believe are socially acceptable.

Our models show a significant association between self-care and quality of life; however, the R^2^ value suggests that other factors not included may also play an important role.

## 5. Conclusions

This study examined the relationship between self-care behaviors and quality of life in individuals newly diagnosed with T2D in Vlore, Albania. The findings highlight the significant impact of self-care activities, such as diet, physical activity, and self-care engagement, on quality of life, especially in the physical and psychological domains.

Our results suggest that improving self-care behaviors could potentially enhance the quality of life for individuals with diabetes, underscoring the importance of structured health education and continuous follow-up. Moreover, age, education status, and BMI emerged as key factors influencing quality of life outcomes, indicating that tailored interventions may be necessary for different patient subgroups.

Promoting self-care practices from the early stages of diagnosis is crucial for newly diagnosed patients in Albania. Tailored educational programs on the importance of diet and regular activity could help improve self-management and prevent future complications.

Finally, health policies should prioritize promoting self-care and ensure targeted support for vulnerable groups, such as older adults and those with limited health literacy. Future research with more extensive and diverse populations and more extended follow-up periods will be essential to confirm and expand upon these findings in the Albanian context.

## Figures and Tables

**Table 1:** Inclusion and exclusion criteria of participants.

	Criteria for Inclusion in the Study	Criteria for Non-Participation
Diagnose	Patients diagnosed with T2D for less than 1 year (newly diagnosed)	Patients diagnosed with type 1 diabetes or gestational diabetes
Age	Patients aged over 18 years and under 75 years	Patients younger than 18 years or older than 75 years
Receiving healthcare	Receiving healthcare at the primary healthcare centers in Vlora	Receiving healthcare in private service as well as in other cities
Presence of chronic pathologies	Diabetic patients who, despite having comorbidities, have the physical and mental capacity to be included in the study	Diabetic patients with severe chronic pathologies that prevent their inclusion in the study
Informed consent	Be willing to participate and have given informed consent	Patients who did not give informed consent to participate
Language and meaning of study materials	Those who speak the language of the study and can understand the study materials.	Those who do not speak the study language or cannot understand the study materials

**Table 2:** Demographic characteristics of participants and pathological profile.

Variables	N = 332	(%)	Mean ± SD
Demographic Characteristics of Participants			
Age (years) (Mean, standard deviation)			66.7 ± 9.1
≤60 years	74	22.3	53.0 ± 5.083
>60 years	258	77.7	70.6 ± 5.473
Gender			
Male	157	47.3	
Female	175	52.7	
Religious belief			
Bektash	16	4.8	
Christian	73	22.0	
Muslim	233	70.2	
No religious affiliation	10	3.0	
Marital status			
Married	293	88.3	
Divorced	13	3.9	
Widowed	22	6.6	
Unmarried	4	1.2	
Educational level			
No primary education	1	0.3	
Incomplete primary education	7	2.1	
Primary education	122	36.8	
High school	176	53.0	
Tertiary education	26	7.8	
Residence			
Rural	57	17.2	
Urban	275	82.8	
Occupation			
Employed	74	22.3	
Retired	216	65.1	
Unemployed	42	12.6	
The patient’s pathological profile			
Diagnosed duration			
Under 3 months	60	18.1	
3–6 months	80	24.1	
6–9 months	116	34.9	
9–12 months	76	22.9	
Family history with diabetes (yes)	62	18.7	
First-degree relatives	55	16.6	
Second-degree relatives	3	0.9	
BMI (baseline)			
Normal	45	13.6	23.6 ± 1.0
Overweight	174	52.4	27.4 ± 1.4
Obesity class I	89	26.8	31.6 ± 2.3
Obesity class II	19	5.7	36.9 ± 1.7
Obesity class III	5	1.5	41.8 ± 1.7
BMI category at moment of interview (kg/m^2^)			
Normal weight	58	17.5	23.6 ± 0.9
Overweight	183	55.1	27.4 ± 1.4
Obesity class I	73	22	31.9 ± 1.5
Obesity class II	16	4.8	36.7 ± 1.3
Obesity class III	2	0.6	42.8 ± 2.8
Systolic blood pressure mmHg			132.9 ± 13.1
Diastolic blood pressure mmHg			82.7 ± 8.1
The glucose level (mg/dL)—baseline			237.7 ± 71.5
The glucose level at the moment of the interview (mg/dL)			184.4 ± 62.7
HbA1c (%)—baseline			8.6 ± 1.6
HbA1c at the moment of interview (%)			8.0 ± 1.4
Lipid profile			
Cholesterol (mg/dL)			221.1 ± 50.5
TG Triglicerid (mg/dL)			197.2 ± 128.0
LDL (mg/dL)			137.2 ± 61.2
HDL (mg/dL)			54.9 ± 35.4
Comorbidity (yes)	160	48.2	
Hypertension (yes)	145	43.7	
Alcohol use (yes)	14	4.2	
How many years?			12.0 ± 9.3
Can you specify the quantity of alcohol you use in mL? (mL/week)			29.3 ± 24.9
Tobacco use (yes)	17	5.1	
How many years?			12.6 ± 7.8
How many cigarettes did you smoke at the time of diagnosis with T2DM? (cigaretes/day)			15.3 ± 8.1
How many cigarettes do you smoke now? (cigaretes/day)			6.2 ± 5.3

**Table 3:** Self-care practices of newly diabetic patients based on SDSCA during the last week.

Self-Care Activity	Mean (No. of Day/Week)	Std. Deviation
**Diet**	3.4	0.79
How many of the last seven days have you followed a healthful eating plan?	4.2	0.74
On average, over the past month, how many days per week have you followed your eating plan?	3.8	0.86
On how many of the last seven days did you eat five or more servings of fruits and vegetables	4.1	0.87
On how many of the last seven days did you eat high-fat foods, such as red meat or full-fat dairy products?	3.2	1.26
On how many of the last seven days did you space carbohydrates evenly throughout the day?	1.7	1.37
**Exercise**	2.5	1.25
On how many of the last seven days did you participate in at least 30 min of physical activity? (total minutes of continuous activity, including walking)	4.2	1.63
On how many of the last seven days did you participate in a specific exercise session (such as swimming, walking, and biking) other than what you do around the house or as part of your work?	0.9	1.189
**Blood sugar testing**	1.5	0.90
On how many of the last seven days did you test your blood sugar?	1.6	0.90
On how many of the last seven days you test your blood sugar the number of times recommended by your healthcare provider?	1.4	0.99
**Foot care**	1.4	0.61
On how many of the last seven days did you check your feet?	1.9	0.99
On how many of the last seven days did you inspect the inside of your shoes?	0.3	0.64
On how many of the last seven days did you wash your feet?	3.8	1.21
On how many of the last seven days did you soak your feet?	0.7	0.81
On how many of the last seven days did you dry between your toes after washing?	1.5	0.61
**Medication**	5.4	0.83
On how many of the last seven days did you take your recommended diabetes medication?	5.5	0.75
On how many of the last seven days did you take your recommended number of diabetes pills?	5.3	0.92
**Smoking**	Mean (No/day)	**Std. Deviation**
How many cigarettes did you smoke on an average day? (tobacco users)	6.2	5.3
No. of cigarettes when they have been diagnosed	15.3	8.1

**Table 4:** Quality of life of participants and their subdomains.

Principal Domain (Mean ± SD)	Subdomain	Mean ± SD
Physical health 35.36 ± 9.48	Activities of daily living	43.15 ± 12.75
	Dependence on medicinal substances and medical aids	42.47 ± 14.40
	Energy and fatigue	28.08 ± 9.31
	Mobility	29.74 ± 11.08
	Pain and discomfort	39.76 ± 22.42
	Sleep and rest	42.77 ± 14.03
	Work capacity	21.54 ± 19.99
Psychological health 34.26 ± 9.23	Bodily image and appearance	39.53 ± 13.80
	Negative feelings	28.76 ± 10.50
	Positive feelings	38.78 ± 14.02
	Self-esteem	39.76 ± 13.62
	Spirituality/religion/personal beliefs	42.84 ± 13.45
	Thinking, learning, memory and concentration	15.36 ± 17.20
Social relationships 40.77 ± 15.93	Personal relationships	46.24 ± 18.23
	Social support	49.59 ± 17.20
	Sexual activity	18.46 ± 22.49
Environment 30.93 ± 9.04	Financial resources	26.05 ± 7.45
	Freedom, physical safety, and security	25.98 ± 6.80
	Health and social care: accessibility and quality	35.39 ± 25.21
	Home environment	30.34 ± 23.87
	Opportunity to acquire new information and skills	28.46 ± 10.06
	Participation in and opportunities for recreation/leisure activities	26.73 ± 7.70
	Physical environment (pollution, noise, traffic, climate)	34.64 ± 18.47
	Transport	26.58 ± 7.97
Overall QoL		35.33 ± 8.25

**Table 5:** The relationship of quality-of-life components with the sociodemographic factors of diabetic patients.

Variables	Physical Health		Psychological Health		Social Relationships		Environmental		Overall	
	Median	*p* Value	Median	*p* Value	Median	*p* Value	Median	*p* Value	Median	*p* Value
	(IQR)		(IQR)		(IQR)		(IQR)		(IQR)	
**Gender**										
Female	31 (7)	0.032 ^a^*	31 (13)	0.700 ^a^	31 (25)	0.096 ^a^	31 (13)	0.003 ^ah^	32.8 (12.8)	0.025 ^a^*
Male	38 (13)		31 (7)		44 (19)		31 (13)		36.3 (11)	
**Age**										
≤60 years	38 (13)	0.051 ^a^	31 (13)	0.538 ^a^	44 (12)	<0.0001 ^ah^	31 (13)	0.445 ^a^	37.5 (14.3)	0.003 ^ah^
>60 years	38 (7)		31 (13)		31 (25)		31 (13)		34.3 (12.3)	
**Occupation**										
Unemployed/retired	38 (7)	0.106 ^a^	31 (13)	0.499 ^a^	31 (25)	0.001 ^ah^	31 (13)	0.879 ^a^	34.4 (12.5)	0.010 ^a^*
Employed	38 (13)		31 (13)		44 (12)		31 (13)		37.5 (16.1)	
**Residence**										
Urban	38 (13)	0.002 ^ah^	31 (13)	0.002 ^ah^	44 (19)	0.961 ^a^	31 (13)	0.001 ^ah^	35.8 (12.8)	0.021 ^a^*
Rural	31 (13)		31 (10)		44 (22)		25 (12)		32.8 (9.8)	
**Education status**										
<Higher education	31 (7)	0.349 ^a^	31 (7)	0.004 ^ah^	31 (25)	0.047 ^a^*	31 (13)	0.823 ^a^	33.6 (9.8)	0.040 ^a^*
Higher/tertiary education	38 (13)		38 (13)		44 (19)		31 (13)		36 (13.1)	
**Civil status**										
Not married	38 (13)	0.231 ^a^	31 (7)	0.467 ^a^	31 (25)	0.118 ^a^	31 (6)	0.193 ^a^	32.8 (15.5)	0.347 ^a^
Married	38 (13)		31 (13)		44 (19)		31 (13)		35.8 (11.3)	
**Religious belief**										
Bektashi	38 (6)	0.045 ^b^*	41 (11.3)	0.057 ^b^	44 (19)	0.732 ^b^	38 (7)	0.024 ^b^*	40 (8.8)	0.069 ^b^
Christian	38 (10)		31 (7)		44 (19)		31 (13)		36 (11.3)	
Muslim	31 (13)		31 (13)		44 (25)		31 (13)		34.3 (12.7)	
No religious affiliation	38 (13)		38 (13)		31 (25)		25 (6)		34.8 (7.8)	

Note: (^a^) Mann–Whitney, (^b^) Kruskal–Wallis, (*) significance < 0.05, (^h^) adjusted significance < 0.007.

**Table 6:** The relationship of quality of life components with patient clinical factors and the use of health services.

Variables	Physical Health		Psychological Health		Social Relationships		Environmental		Overall	
	Median (IQR)	*p* Value	Median (IQR)	*p* Value	Median (IQR)	*p* Value	Median (IQR)	*p* Value	Median (IQR)	*p* Value
**Duration of diabetes**										
<3 months	31 (13)	0.045 ^b^*	31 (10.5)	0.050 ^b^	38 (19)	0.608 ^b^	25 (4.5)	0.037 ^b^*	29.8 (7.4)	0.084 ^b^
3–6 months	38 (7)		31 (13)		31 (19)		31 (6)		32.8 (11)	
6–9 months	38 (13)		38 (13)		44 (25)		31 (13)		37.5 (12.5)	
9–12 months	44 (6)		38 (13)		50 (25)		31 (19)		40 (10.7)	
**Comorbidity**	31 (7)	<0.0001 ^ah^	31 (11.5)	0.063 ^a^	44 (25)	0.851 ^a^	31 (6)	0.006 ^a^**	36 (12.5)	0.053 ^a^
No comorbidity	38 (13)		31 (13)		44 (19)		31 (13)		34.3 (11)	
**HTN**	31 (13)	0.001 ^ah^	31 (13)	0.031 ^a^*	31 (25)	0.089 ^a^	31 (13)	0.295 ^a^	32.8 (9.5)	0.010 ^a^*
No HTN	38 (13)		31 (7)		44 (19)		28 (13)		35.9 (12.8)	
**Changes in BMI**										
Reduction >1%	38 (13)	<0.0001 ^bh^	38 (13)	<0.0001 ^bh^	44 (25)	0.001 ^bh^	31 (13)	<0.0001 ^bh^	37.5 (11)	<0.0001 ^bh^
No changes	31 (6)		31 (1)		31 (19)		25 (6)		28 (8)	
Increase >1%	31 (7)		31 (7)		31 (25)		25 (6)		32.8 (10)	
**Changes in HbAc1**										
Reduction	38 (13)	<0.0001 ^ah^	38 (13)	<0.0001 ^ah^	44 (19)	0.019 ^a^*	31 (13)	<0.0001 ^ah^	37.3 (11)	<0.0001 ^ah^
Increase/no changes	31 (13)		31 (11.5)		31 (25)		25 (12)		29.8 (8.9)	
**Level of HbA1c <** **7%**	38 (6)	<0.0001 ^ah^	44 (6)	<0.0001 ^ah^	44 (25)	0.016 ^a^*	34.5 (13)	<0.0001 ^ah^	41 (9.5)	<0.0001 ^ah^
No	31 (7)		31 (7)		44 (25)		25 (13)		33 (11.3)	
**Doctor visits: How often do you see an endocrinologist?**										
Never	31 (19)	0.109 ^b^	31 (15.5)	0.050 ^b^	25 (25)	0.051 ^b^	25 (6)	0.003 ^bh^	28 (16.3)	0.008 ^b^*
Once a year	38 (13)		31 (7)		44 (19)		31 (13)		35.8 (12.5)	
Twice a year	38 (13)		31 (13)		44 (25)		31 (13)		34.5 (11)	
Three times a year	44 (14.5)		41 (14.5)		50 (28.3)		38 (7.5)		42.4 (13.2)	
**How often do you see your family doctor?**										
Every month	38 (13)	0.072 ^b^	31 (13)	0.958 ^b^	44 (19)	0.421 ^b^	31 (13)	0.058 ^b^	35.8 (11)	0.537 ^b^
Every three months	31 (10)		31 (13)		44 (25)		25 (6)		36 (9.4)	
Once in six months	31 (19)		31 (7)		31 (37)		25 (12)		28 (14.3)	
Once a year	31 (20)		38 (22)		31 (13)		28 (20.3)		35.1 (12.8)	
Every two months	31 (19)		31 (19)		44 (26.5)		28 (6)		34.4 (14.6)	
Every four months	44 (13)		31 (0)		44 (0)		25 (0)		32.8 (0)	
**Have you been to the ophthalmologist?**										
No	31 (7)	0.001 ^ah^	31 (7)	0.001 ^ah^	44 (25)	0.012 ^a^*	31 (13)	0.035 ^a^*	33 (11)	<0.0001 ^ah^
Yes	38 (13)		38 (13)		44 (19)		31 (13)		37.8 (11.4)	
**Hospitalization**										
No	38 (13)	0.309 ^b^	31 (13)	0.391 ^b^	44 (19)	0.385 ^b^	31 (13)	0.704 ^b^	34.5 (11.3)	0.885 ^b^
1 time	31 (22)		31 (19)		31 (31)		31 (22)		33 (19.6)	
2 times	37.5 (0)		37.5 (0)		50 (0)		31.5 (0)		39.1 (0)	

Note: (^a^) Mann–Whitney, (^b^) Kruskal–Wallis, (*) significance < 0.05, (**) significance <0.01, (^h^) adjusted significance < 0.005.

**Table 7:** Association of quality-of-life components with patients’ self-care.

Self-Care and Its Components	Physical Health		Psychological Health		Social Relationships		Environmental		Overall	
	Median (IQR)	*p* Value	Median (IQR)	*p* Value	Median (IQR)	*p* Value	Median (IQR)	*p* Value	Median (IQR)	*p* Value
**Overall self-care (active)**	38 (13)	<0.0001 ^h^	44 (6)	<0.0001 ^h^	50 (25)	0.001 ^h^	38 (13)	<0.0001 ^h^	40.8 (10.1)	<0.0001 ^h^
Non-active	31 (7)		31 (7)		44 (25)		25 (13)		33 (11.3)	
**Dietary self-care (active)**	38 (13)	<0.0001 ^h^	38 (13)	<0.0001 ^h^	44 (25)	0.100	31 (13)	<0.0001 ^h^	37.5 (11)	<0.0001 ^h^
Non-active	31 (13)		31 (7)		44 (25)		25 (13)		32.8 (11.3)	
**Physical activity self-care (active)**	38 (13)	<0.0001 ^h^	38 (13)	<0.0001 ^h^	50 (25)	<0.0001 ^h^	31 (13)	<0.0001 ^h^	39.3 (11.3)	<0.0001 ^h^
Non-active	31 (7)		38 (13)		31 (25)		25 (13)		33 (11.3)	
**Medication self-care (active)**	38 (13)	-	31 (13)	-	44 (19)	-	31 (13)	-	34.5 (11.3)	-
Non-active	0		0		0		0		0	
**Glucose monitoring self-care (active)**	38 (13)	0.563	31 (13)	0.611	44 (19)	0.930	31 (13)	0.409	34.3 (11.3)	0.235
Non-active	38 (13)		31 (10)		44 (25)		31 (13)		35.8 (12.6)	
**Foot care self-care (active)**	38 (13)	0.375	31 (7)	<0.0001 ^h^	44 (25)	0.606	31 (10)	0.011 *	37.8 (12)	0.039 *
Non-active	31 (22)		44 (3)		31 (16)		31 (13)		34.5 (12.5)	

Note: Statistical test: Mann–Whitney U, (*) significance < 0.05, (^h^) adjusted significance < 0.008.

**Table 8:** Multiple linear regression analysis of factors influencing overall and component quality of life in diabetic patients.

Variables	Category	Physical Health		Psychological Health		Social Relationships		Environmental		Overall	
		B 95% CI	*p*	B 95% CI	*p*	B 95% CI	*p*	B 95% CI	*p*	B 95% CI	*p*
Gender	Female/Male (Ref)							−2.49 [−4.34; −0.648]	0.008		
Age	>60 years/≤60 years (Ref)					−6.61 [−11.63; −1.59]	0.010			−4.36 [−6.29; −2.42]	<0.0001
Education status	≤High education/Tertiary education (Ref)			−3.09 [−6.49; 0.31]	0.075						
Occupation	Unemployed/Employed (Ref)					−6.54 [−11.55; −1.52]	0.011	−3.41 [−6.02; −0.80]	0.011		
Residence	Rural/Urban (Ref)	−1.21 [−3.81; 1.38]	0.360	−1.91 [−4.48; 0.66]	0.144						
Comorbidity	Comorbidity/No comorbidity	−4.32 [−6.30; −2.35]	<0.0001								
Changes in BMI	Increase BMI > 1%/reduction, no changes (Ref)	−3.04 [−4.67; −1.41]	<0.0001	−2.28 [−3.90; −0.66]	0.004	−5.12 [−7.85; −2.38]	<0.0001	−2.54 [−4.15; −0.92]	0.002	−3.33 [−4.74; −1.92]	<0.0001
Changes in HbA1c	Increase HbA1c/reduction, no changes (Ref)	−5.28 [−7.48; −3.09]	<0.0001	−4.59 [−6.76; −2.41]	<0.001			−4.05 [−6.24; −1.85]	<0.001	−4.75 [−6.67; −2.84]	<0.001
HTA	Having HTA/No HTA (Ref)	−1.13 [−4.41; 2.14]	0.494								
How often do you seen an endocrinologist	Never or 1 time/year/More often (Ref)					−0.44 [−1.55; 2.42]	0.667				
Have you been to the ophthalmologist	No/Yes (Ref)	−2.83 [−4.82; −0.84]	0.005	−2.59 [−4.56; −0.64]	0.010						

**Table 9:** Multiple linear regression analysis of the impact of self-care elements on the quality of life in diabetic patients.

Variables	Category	Physical Health		Psychological Health		Social Relationships		Environmental		Overall	
		B 95% CI	*p*	B 95% CI	*p*	B 95% CI	*p*	B 95% CI	*p*	B 95% CI	*p*
Dietary Self−care	No active/Active (Ref)	−4.74 [−6.78; −2.72]	<0.0001	−3.63 [−5.57; −1.70]	<0.0001			−3.73 [−5.67; −1.79]	<0.0001	−3.82 [−5.55; −2.08]	<0.0001
Physical activity self−care	No active/Active (Ref)	−4.32 [−6.66; −1.98]	<0.0001	−3.35 [−5.64; −1.06]	0.004	−9.27 [−13.25; −5.30]	<0.0001	−4.80 [−7.03; −2.55]	<0.0001	−5.45 [−7.44; −3.46]	<0.0001
Foot care self−care	No active/Active (Ref)			−8.55 [−13.53; −3.57]	0.001						

**Table 10:** Simple linear regression analysis of the impact of overall self-care on the quality of life in diabetic patients.

Variables	Category	Physical Health		Psychological health		Social Relationships		Environmental		Overall	
		B 95% CI	*p*	B 95% CI	*p*	B 95% CI	*p*	B 95% CI	*p*	B 95% CI	*p*
Overall Self−care	No active/Active (Ref)	−7.05 [−9.51; −4.58]	<0.0001	−9.25 [−11.49; −6.99]	<0.0001	−7.01 [−11.27; −2.73]	0.001	−7.15 [−9.48; −4.81]	<0.0001	−7.65 [−9.74; −5.55]	<0.0001

## Data Availability

The authors will make the raw data supporting this article’s conclusions available upon request.

## Public Involvement Statement

There was no public involvement in this research.

## Guidelines and Standards Statement

This manuscript was prepared following the STROBE guidelines for observational research. The complete STROBE checklist and reporting guidelines are available via the EQUATOR Network: https://www.strobe-statement.org/ (accessed on 20 January 2025).
